# Supplementary material for: Epidemiological Characteristics and Spatiotemporal Clustering of Symptomatic Hepatitis E Virus Reinfection in Zhejiang Province, 2005–2023
Source: Viruses. 2024 Oct 26;16(11):1676. doi: 10.3390/v16111676 (PMC11599083; doi:10.3390/v16111676)
Supplement: Supplementary file 1 [file viruses-16-01676-s001.zip › Table S1-S6.pdf]

Table S1. Demographic Characteristics of Hepatitis E Infected Individuals in Hangzhou.

	Single infection (n=13487)	Reinfection		
		Overall (n=156)	Infected twice (n=148)	Three times (n=8)
Gender				
Male	8858 (65.7)	94 (60.3)	89 (60.1)	5 (62.5)
Female	4629 (34.3)	62 (39.7)	59 (39.9)	3 (37.5)
Age at first infection	49.0 (37.0, 60.0)	46.0 (36.0, 57.0)	46.0 (36.0, 57.0)	48.5 (31.5, 56.8)
Age at first reinfection	-	50.0 (38.0, 61.0)	46.0 (38.0, 61.0)	52.5 (32.8, 59.3)
Occupation				
Farmers	4456 (33.0)	39 (25.0)	-	-
Workers in service industry	1742 (12.9)	27 (17.3)	-	-
Manual workers	2002 (14.8)	20 (12.8)	-	-
Persons with unemployment	866 (6.4)	10 (6.4)	-	-
Retired persons	1639 (12.2)	24 (15.4)	-	-
Office workers	1032 (7.7)	24 (15.4)	-	-
Workers in the catering industry	154 (1.1)	3 (1.9)	-	-
Healthcare workers	101 (0.7)	0 (0.0)	-	-
Students	132 (1.0)	1 (0.6)	-	-
School teachers	167 (1.2)	1 (0.6)	-	-
Unknown	1196 (8.9)	7 (4.5)	-	-

Table S2. Demographic Characteristics of Hepatitis E Infected Individuals in Wenzhou.

	**Single infection (n=4897)**	**Reinfection**		
		**Overall (n=77)**	**Infected twice (n=72)**	**Three times (n=5)**
Gender				
Male	3354 (68.5)	42 (54.5)	39 (54.2)	3 (60.0)
Female	1543 (31.5)	35 (45.5)	33 (45.8)	2 (40.0)
Age at first infection	48.0 (36.0, 59.0)	46.0 (36.5, 57.5)	46.0 (37.5, 56.8)	40.0 (17.5, 65.0)
Age at first reinfection	-	49.0 (38.5, 59.0)	49.5 (39.5, 59.5)	41.0 (18.5, 66.0)
Occupation				
Farmers	901 (18.4)	12 (15.6)	-	-
Workers in service industry	295 (6.0)	14 (18.2)	-	-
Manual workers	997 (20.4)	9 (11.7)	-	-
Persons with unemployment	701 (14.3)	13 (16.9)	-	-
Retired persons	188 (3.8)	1 (1.3)	-	-
Office workers	155 (3.2)	2 (2.6)	-	-
Workers in the catering industry	168 (3.4)	3 (3.9)	-	-
Healthcare workers	29 (0.6)	1 (1.3)	-	-
Students	67 (1.4)	0 (0.0)	-	-
School teachers	42 (0.9)	0 (0.0)	-	-
Unknown	1354 (27.6)	22 (28.6)	-	-

Table S3. Demographic Characteristics of Hepatitis E Infected Individuals in Taizhou.

	**Single infection (n=4417)**	**Reinfection**	
		**Overall (n=72)**	**Infected twice (n=71)**
Gender			
Male	2866 (64.9)	42 (58.3)	41 (57.7)
Female	1551 (35.1)	30 (41.7)	30 (42.3)
Age at first infection	49.0 (38.0, 59.0)	45.0 (36.0, 49.8)	45.0 (36.0, 50.0)
Age at first reinfection	-	49.0 (41.0, 55.8)	49.0 (41.0, 55.5)
Occupation			
Farmers	2815 (63.7)	32 (44.4)	-
Workers in service industry	122 (2.8)	8 (11.1)	-
Manual workers	291 (6.6)	6 (8.3)	-
Persons with unemployment	351 (7.9)	11 (15.3)	-
Retired persons	123 (2.8)	3 (4.2)	-
Office workers	133 (3)	2 (2.8)	-
Workers in the catering industry	84 (1.9)	6 (8.3)	-
Healthcare workers	17 (0.4)	2 (2.8)	-
Students	64 (1.4)	0 (0)	-
School teachers	46 (1)	0 (0)	-
Unknown	371 (8.4)	2 (2.8)	-

Table S4. Number of initial HEV infections cases per 1,000,000 population in various cities of Zhejiang Province, 2005-2023.

City	2005	2006	2007	2008	2009	2010	2011	2012	2013	2014	2015	2016	2017	2018	2019	2020	2021	2022	2023
Hangzhou	55.815	61.958	65.887	61.637	61.358	68.352	100.824	107.589	74.062	81.759	89.599	109.382	94.423	60.778	56.661	40.201	67.191	77.489	90.902
Ningbo	22.107	39.309	38.724	36.917	26.426	26.015	19.664	17.803	19.314	20.100	21.981	15.873	20.862	29.073	14.633	14.544	24.413	22.978	31.399
Wenzhou	33.175	34.350	34.299	24.756	23.403	24.849	29.531	21.298	19.028	22.276	18.208	15.368	25.719	26.675	34.515	12.830	42.716	54.551	54.758
Jiaxing	24.024	30.392	25.084	24.811	26.902	29.523	42.154	25.968	31.154	28.446	22.246	22.757	23.411	18.543	23.618	14.230	27.194	25.401	25.401
Huzhou	54.820	54.210	59.286	62.766	45.965	53.214	50.707	25.818	26.749	17.406	21.017	23.866	27.045	24.241	21.942	12.456	28.764	24.319	30.472
Shaoxing	15.490	18.008	18.773	26.316	24.027	37.655	38.103	26.300	30.915	23.406	15.298	11.227	14.571	10.147	14.449	9.639	20.798	15.319	22.044
Jinhua	31.076	46.731	38.235	30.091	27.079	27.022	47.345	24.264	25.608	16.001	11.918	11.957	11.862	13.864	11.431	8.921	21.910	25.958	28.483
Quzhou	27.753	60.606	59.009	54.284	67.114	54.169	60.878	46.226	63.559	54.614	43.601	37.928	44.851	45.941	48.608	36.874	41.539	59.389	38.428
Zhoushan	29.268	44.747	26.087	26.565	12.230	8.029	12.313	17.544	3.503	6.981	6.076	2.591	5.137	6.820	5.150	4.314	24.893	19.658	16.239
Taizhou	40.450	41.367	49.355	43.350	48.480	44.024	44.341	27.977	22.855	32.086	31.575	40.789	57.862	32.830	32.529	20.824	39.033	40.132	44.774
Lishui	23.841	46.134	34.558	30.435	45.474	51.936	75.142	52.433	34.873	30.971	14.493	14.781	22.415	15.957	17.778	12.360	23.469	24.652	27.435

Table S5. Number of second HEV infections cases per 1,000,000 population in various cities of Zhejiang Province, 2005-2023.

City	2005	2006	2007	2008	2009	2010	2011	2012	2013	2014	2015	2016	2017	2018	2019	2020	2021	2022	2023
Hangzhou	0.000	0.000	0.127	0.251	0.370	0.345	0.916	0.341	1.018	1.237	1.885	1.197	1.584	1.511	0.947	0.669	0.983	1.131	0.889
Ningbo	0.000	0.000	0.000	0.283	0.000	0.131	0.262	0.131	0.130	0.128	0.383	0.254	0.500	0.110	0.215	0.637	0.524	0.104	0.728
Wenzhou	0.000	0.128	0.253	0.000	0.248	0.109	0.000	0.109	0.109	0.221	0.110	0.545	0.543	0.945	0.941	1.252	0.829	0.723	1.136
Jiaxing	0.000	0.000	0.000	0.000	0.000	0.000	0.000	0.000	0.219	0.000	0.000	0.000	0.000	0.191	0.000	0.185	0.363	0.540	0.540
Huzhou	0.000	0.000	0.000	0.355	0.351	0.000	0.345	0.344	0.686	0.683	0.678	0.000	0.368	0.307	0.000	0.297	0.294	0.000	0.293
Shaoxing	0.000	0.000	0.000	0.216	0.000	0.000	0.608	0.405	0.202	0.000	0.201	0.200	0.000	0.000	0.190	0.000	0.375	0.187	0.187
Jinhua	0.000	0.000	0.000	0.194	0.376	0.000	0.000	0.370	0.000	0.368	0.000	0.181	0.180	0.147	0.289	0.142	0.000	0.281	0.421
Quzhou	0.000	0.000	0.000	0.000	0.000	0.371	0.000	0.372	0.742	0.371	0.738	0.000	0.358	0.000	1.026	0.578	0.237	1.010	0.673
Zhoushan	0.000	0.000	0.000	0.000	0.000	0.000	0.000	0.000	0.000	0.000	0.000	0.000	0.000	0.000	0.000	0.000	0.858	0.000	0.000
Taizhou	0.000	0.000	0.000	0.000	0.174	0.167	0.333	0.167	0.000	0.831	0.496	1.316	0.654	0.925	0.611	0.905	1.802	1.348	1.497
Lishui	0.000	0.000	0.437	0.000	0.000	0.472	0.000	0.000	0.000	0.000	0.000	0.000	0.457	0.000	0.000	0.000	0.398	0.000	0.398

Table S6. Number of third HEV infections cases per 1,000,000 population in various cities of Zhejiang Province, 2005-2023.

City	2005	2006	2007	2008	2009	2010	2011	2012	2013	2014	2015	2016	2017	2018	2019	2020	2021	2022	2023
Hangzhou	0.000	0.000	0.000	0.000	0.000	0.000	0.000	0.000	0.000	0.000	0.111	0.000	0.000	0.089	0.086	0.167	0.082	0.000	0.000
Ningbo	0.000	0.000	0.000	0.000	0.000	0.000	0.000	0.000	0.000	0.000	0.000	0.000	0.000	0.000	0.000	0.000	0.000	0.000	0.000
Wenzhou	0.000	0.000	0.000	0.000	0.000	0.000	0.109	0.000	0.000	0.000	0.000	0.000	0.000	0.000	0.209	0.104	0.104	0.000	0.000
Jiaxing	0.000	0.000	0.000	0.000	0.000	0.000	0.000	0.000	0.000	0.000	0.000	0.000	0.000	0.000	0.000	0.000	0.000	0.000	0.000
Huzhou	0.000	0.000	0.000	0.000	0.000	0.000	0.000	0.000	0.000	0.000	0.339	0.000	0.000	0.000	0.000	0.000	0.000	0.000	0.000
Shaoxing	0.000	0.000	0.000	0.000	0.000	0.000	0.000	0.000	0.000	0.000	0.000	0.000	0.000	0.000	0.000	0.000	0.000	0.000	0.000
Jinhua	0.000	0.000	0.000	0.000	0.000	0.000	0.000	0.000	0.000	0.000	0.000	0.000	0.000	0.000	0.000	0.000	0.000	0.000	0.000
Quzhou	0.000	0.000	0.000	0.000	0.000	0.000	0.000	0.000	0.000	0.000	0.000	0.000	0.000	0.000	0.000	0.439	0.000	0.000	0.000
Zhoushan	0.000	0.000	0.000	0.000	0.000	0.000	0.000	0.000	0.000	0.000	0.000	0.000	0.000	0.000	0.000	0.000	0.000	0.000	0.000
Taizhou	0.000	0.000	0.000	0.000	0.000	0.000	0.000	0.000	0.000	0.000	0.000	0.000	0.000	0.000	0.000	0.151	0.000	0.000	0.000
Lishui	0.000	0.000	0.000	0.000	0.000	0.000	0.000	0.000	0.000	0.000	0.000	0.000	0.000	0.000	0.000	0.000	0.000	0.000	0.000
